# MicroRNA Transcriptomes Relate Intermuscular Adipose Tissue to Metabolic Risk

**DOI:** 10.3390/ijms14048611

**Published:** 2013-04-22

**Authors:** Jideng Ma, Shuzhen Yu, Fengjiao Wang, Lin Bai, Jian Xiao, Yanzhi Jiang, Lei Chen, Jinyong Wang, Anan Jiang, Mingzhou Li, Xuewei Li

**Affiliations:** 1 Institute of Animal Genetics & Breeding, College of Animal Science & Technology, Sichuan Agricultural University, Ya’an 625014, China; E-Mails: jideng_ma@sina.com (J.M.); yushuzhen1988@126.com (S.Y.); wangfengjiaosicau@gmail.com (F.W.); blin16@126.com (L.B.); jianxiao112@163.com (J.X.); lingdang317@163.com (A.J.); 2 College of Life and Basic Sciences, Sichuan Agricultural University, Ya’an 625014, China; E-Mail: jiangyz04@163.com; 3 Chongqing Academy of Animal Science, Chongqing 402460, China; E-Mails: sicau.chen@gmail.com (L.C.); kingyou@vip.sina.com (J.W.)

**Keywords:** intermuscular adipose tissue (IMAT), metabolic risk, miRNA, pig, immune response, inflammation response, obesity, transcriptome

## Abstract

Intermuscular adipose tissue is located between the muscle fiber bundles in skeletal muscles, and has similar metabolic features to visceral adipose tissue, which has been found to be related to a number of obesity-related diseases. Although various miRNAs are known to play crucial roles in adipose deposition and adipogenesis, the microRNA transcriptome of intermuscular adipose tissue has not, until now, been studied. Here, we sequenced the miRNA transcriptomes of porcine intermuscular adipose tissue by small RNA-sequencing and compared it to a representative subcutaneous adipose tissue. We found that the inflammation- and diabetes-related miRNAs were significantly enriched in the intermuscular rather than in the subcutaneous adipose tissue. A functional enrichment analysis of the genes predicted to be targeted by the enriched miRNAs also indicated that intermuscular adipose tissue was associated mainly with immune and inflammation responses. Our results suggest that the intermuscular adipose tissue should be recognized as a potential metabolic risk factor of obesity.

## 1. Introduction

Adipose tissues (ATs) play a vital role in energy homeostasis and process the largest energy reserve in the body of animals. The rapidly expanding adipokine family is secreted by ATs [1], and, as a result, AT has been identified as an endocrine organ that influences a variety of physiological and pathological processes (such as immunity and inflammation) [2,3] that are involved in the development of metabolic diseases such as cardiovascular disease and type 2 diabetes mellitus [4–6]. Functional and metabolic differences between the visceral and subcutaneous ATs have been well documented. Subcutaneous AT mainly affects metabolic processes, while visceral AT has been identified as a metabolic risk factor for obesity. Recent studies have revealed that the intermuscular adipose tissue (IMAT), which is located between the muscle fiber bundles in skeletal muscles, has similar functional and metabolic features as the visceral ATs [7,8]. Indeed, IMAT was found in greater amounts than visceral AT in acromegaly patients despite their increased muscle mass, suggesting that increased amounts of AT in muscles might be associated with growth hormone-induced insulin resistance [9].

MicroRNAs (miRNAs) are endogenous small non-coding RNAs that modulate gene expression at a post-transcriptional level by binding to the 3′ untranslated region (3′-UTR) of the target mRNAs [10]. During the past decade, various miRNAs that play crucial roles in adipose deposition and adipogenesis have been identified. Typically, miR-143 was identified as a pro-adipogenic modulator during pre-adipocyte differentiation [11,12]. MiR-103 [13] and the miR-17-92 cluster [14] were reported to accelerate adipocyte differentiation. MiR-27a [15], miR-27b [16], miR-448 [17] and miR-15a [18] were demonstrated to suppress adipogenic differentiation. MiR-519d [19], miR-335 [20] and miR-377 [21] were associated with lipid metabolism disorders. However, features of the miRNA transcriptome of IMAT have yet to be investigated.

*Sus scrofa* (pig) is emerging as an ideal biomedical model for obesity and metabolic disorders in human because of the similarity in metabolic features and proportional organ sizes in these two species [22]. To decipher the unique metabolic and functional features of IMAT, we sequenced the miRNA transcriptomes of porcine IMAT by small RNA-sequencing and compared it with a representative subcutaneous adipose tissue, superficial abdominal subcutaneous adipose tissue (sASAT). We identified various known, conserved, and putative novel porcine miRNAs in these two tissues. Notably, the sASAT-enriched miRNAs were related mainly to lipid metabolic homeostasis, while the IMAT-enriched miRNAs were related mainly to inflammation and diabetes, and the target genes of the IMAT-enriched miRNAs were primarily associated with inflammatory and diabetes processes. Together, these findings indicated the metabolic risk of IMAT. Our results will contribute to studies into the role of IMAT in obesity-related metabolic disease.

## 2. Results and Discussion

### 2.1. Transcriptome Sequencing Data

**Figure 1:**
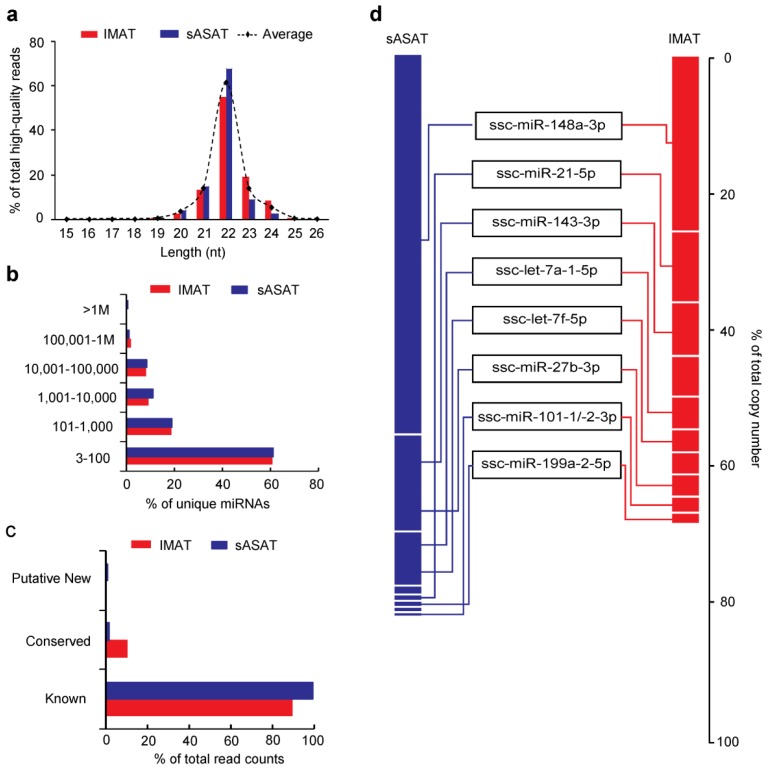
Description of miRNAs in two adipose tissues. (**a**) Length distribution of the high-quality reads; (**b**) Distribution of read counts of the identified miRNAs; (**c**) Distribution of read counts in the three defined miRNA groups; (**d**) Copy numbers of the top 10 miRNAs with highest read counts. IMAT, intermuscular adipose tissue; sASAT, superficial abdominal subcutaneous adipose tissue.

We used a small RNA-sequencing approach to sequence the miRNA transcriptomes of porcine IMAT and sASAT and obtained 17.76 million (M) and 18.50 M raw reads, respectively. More than 80% of the raw reads passed the quality filters (see Methods) and were termed the high-quality reads (IMAT: 14.93 M, 84.09%; sASAT: 14.92 M, 81.59%) (Supplementary Table S1). The high-quality reads in both transcriptomes exhibited the canonical size range distribution that is common to mammalian miRNAs (Figure 1a). The vast majority of the reads were 21–23 nucleotides (nt) in length. The 22-nt reads accounted for 61.50% of all the high-quality reads, followed by the 21-nt (14.00%) and 23-nt (13.99%) reads. This result indicated the reliability of using the small RNA-sequencing approach to generate miRNA reads as candidates for further analysis.

### 2.2. MiRNA Profiling of sASAT and IMAT

A total of 597 mature miRNAs corresponding to 453 miRNA precursors (pre-miRNAs) were identified in the two libraries by mapping them to the pig genome. In agreement with previous reports [23,24], we found that all the miRNA classes consisted of multiple mature variants (the isomiRs). The most abundant isomiR in each class was picked as the reference sequence for that class [23] based on the evidence that there was a significant positive correlation between the counts of most abundant isomiR and the total counts of all isomiRs in the same class (IMAT: Spearman’s *r* = 0.98, *p* < 10^−5^; sASAT: Spearman’s *r* = 0.97, *p* < 10^−4^).

The identified mature miRNAs and their precursors were divided into three subgroups according to alignment criteria (Supplementary Table S2) as: (1) Porcine known miRNAs: 297 miRNAs mapped to 176 known porcine pre-miRNAs; specifically, 210 were in miRBase 18.0 [25] and 87 were novel miRNA*s; (2) Porcine conserved miRNAs: 107 miRNAs mapped to 71 other known mammalian pre-miRNAs in miRBase 18.0 and these pre-miRNAs mapped to the pig genome. These miRNAs were labeled with the names of the corresponding conserved miRNAs; (3) Porcine putative new miRNAs: 230 miRNAs (longer than 18 nt and unmapped to any known mammalian pre-miRNAs in miRBase 18.0) encompassing 206 candidate pre-miRNAs that were predicted RNA hairpins derived from the pig genome, and were labeled PPN (Porcine putative new). Notably, there are the distinct pre-miRNAs coding the identical mature miRNAs, which resulting in 617 miRNAs (*i.e.*, reference sequence) corresponding to 597 unique miRNA sequences (Supplementary Table S3).

The identified miRNAs exhibited a large dynamic range of read counts ranging from 3 to millions. The vast majority of miRNAs (IMAT: 61.11%; sASAT: 61.03%) were in low abundance (3 to 100 read counts) and belonged mainly to the porcine conserved and putative new miRNA groups. Only a few miRNAs (IMAT: 2.11%; sASAT: 1.17%) were in high abundance (>100,000 read counts) and they belonged mainly to the porcine known miRNA group (Figure 1b,c). This result suggests that the low-abundance conserved and putative new miRNAs may have escaped from previous detection efforts.

We found that the top ten miRNAs with the highest abundance contributed 67.46% and 82.94% of the total counts in the IMAT and sASAT libraries, respectively, and eight miRNAs were shared by two libraries in the top 10 positions (Figure 1d). The high abundance of these miRNAs implies that they may have housekeeping cellular roles and may be the main regulatory miRNAs in adipogenesis [11,26,27] and cellular basal metabolism [28,29]. For example, let-7a-5p [12], miR-148a-3p [26], miR-21-5p [27], miR-143-3p [30] and miR-101-3p [13] have been reported to be up-regulated during 3T3-L1 pre-adipocyte differentiation, whereas miR-27b-3p was found to be down-regulated during adipogenesis of human multipotent adipose-derived stem cells [31] and miR-199a-5p was up-regulated in subcutaneous AT in obese versus non-obese individuals [13].

### 2.3. Inflammation- and Diabetes-Related miRNAs Enriched in IMAT

**Figure 2:**
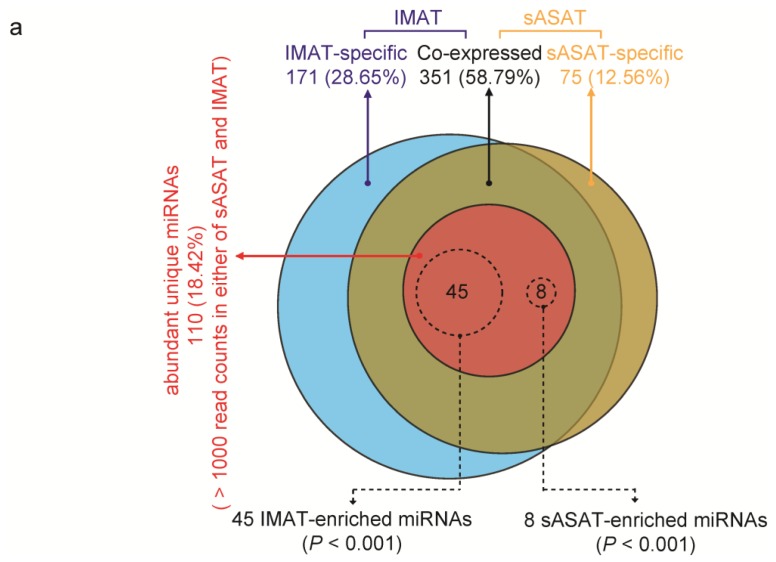

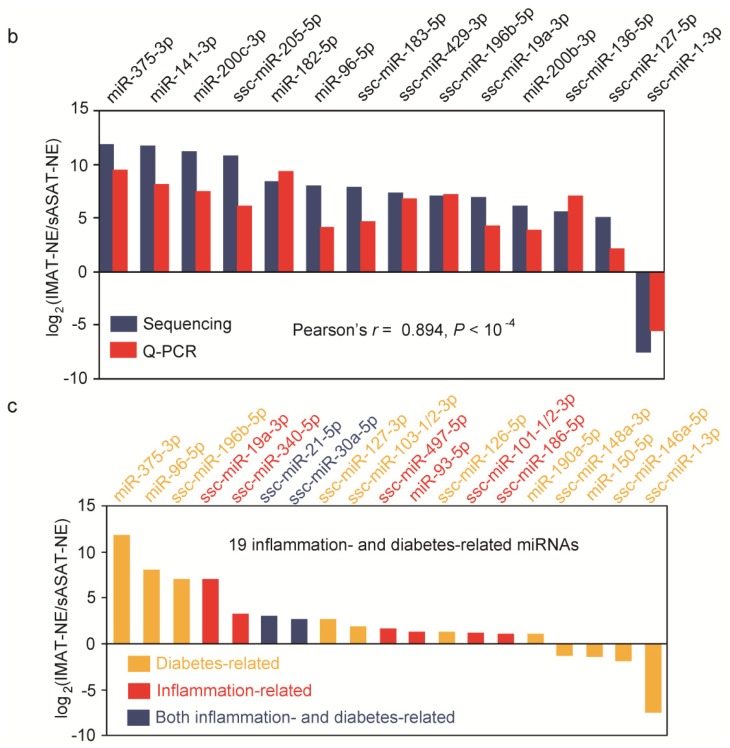
Characteristics of the differentially expressed (DE) miRNAs between porcine sASAT and IMAT. (**a**) Distribution of 597 unique miRNAs between sASAT (blue) and IMAT (yellow). The red circle represents the 110 miRNAs with read counts >1000 in either of the two libraries. The dashed circles indicate the 45 IMAT-enriched (**left**) and eight sASAT-enriched (**right**) miRNAs (*p* < 0.001); (**b**) q-PCR validation for the top 14 DE miRNAs with highest read counts between IMAT and sASAT. Pearson’s correlation was used to determine the relationship between the q-PCR and small RNA-seq results for miRNA expression levels. IMAT-NE and sASAT-NE represent normalized expression levels for the miRNAs in the IMAT and sASAT libraries, respectively; (**c**) The differential expression of 19 inflammation- and diabetes-related miRNAs between IMAT and sASAT.

More than half of the unique miRNAs (351 of 597, 58.79%) were co-expressed in IMAT and sASAT. Only 171 (28.64%) and 75 (12.56%) of the unique miRNAs were expressed specifically in IMAT and sASAT, respectively (Figure 2a and Supplementary Table S3). It was well-known that miRNAs function in a dose-dependent manner [32], thus the less abundant miRNAs (<1000 read counts in both libraries) were considered to be less important and were filtered out. Of the 110 more abundant unique miRNAs (>1000 read counts in either library), 53 (48.18%) were determined to be differentially expressed (DE) between IMAT and sASAT using the IDEG6 program [33] (Figure 2a and Supplementary Table S4). The changes in expression patterns of the top 14 DE miRNAs with the highest read counts showed significant positive correlations between the q-PCR results and the small RNA-sequencing data (Person’s *r* = 0.894, *p* < 10^−4^), again highlighting the reliability of the small RNA-sequencing approach (Figure 2b). Moreover, in the process of q-PCR validation, we also found that all expression levels of selected miRNAs obtained by q-PCR within the biological replicates were highly correlated and with very low deviation, which not only indicated the high repeatability and reliability of the q-PCR approach but also reflected the high purity of our experimental samples (Supplementary Table S5).

Notably, many of the DE miRNAs (19 out of 53, 35.84%) were associated with inflammation and diabetes based on the annotations assigned using the Pathway Central database (SA Biosciences, Frederick, MD, USA) (Figure 2c). Eight inflammation-related and 9 diabetes-related miRNAs were present in higher abundance in the IMAT transcriptome compared with the sASAT transcriptome (Figure 2c). MiR-21 was found to be over-expressed at the inflammation site [34] and it has been suggested that miR-21 could act as a biomarker for inflammation in the aging process and cardiovascular disease [35]. MiR-101 was reported to be related to inflammation and chondrocyte extracellular matrix degradation [36]. Circulating miR-30a was up-regulated in diabetes patients and has been associated with insulin resistance [20]. The ectopic high expression of miR-103 could induce impaired glucose homeostasis or, conversely, the silencing of miR-103 could improve glucose homeostasis and insulin sensitivity [37].

In addition, various IMAT-enriched miRNAs related to pathological responses were found (Supplementary Table S4). For example, miR-182 and miR-183 (members of the miR-183-96-182 cluster) are well-characterized oncomiRs that can promote the clonal expansion of activated helper T lymphocytes [38,39]. MiR-200b, miR-200c and miR-141 (members of the miR-200 family) were reported to be significantly altered in bladder [40] and breast cancers [41]. MiR-191 was suggested as a biomarker for the diagnosis and prognosis of acute myeloid leukemia [42]. These results suggest that IMAT is associated mainly with inflammation- and diabetes-related responses, and should be deemed as a potential metabolic risk factor of obesity.

In contrast, the sASAT-enriched miRNAs (Supplementary Table S4) were mainly related to adipogenesis and lipid metabolism. For example, miR-378 was up-regulated in adipogenesis of human AT-derived stromal cells [43] and the over-expression of miR-378 in ST2 cell line was reported to promote lipid accumulation by enhancing *de novo* lipogenesis [44]. MiR-365 was revealed as a central regulator of brown fat differentiation and adipogenesis [45]. MiR-146a regulated mainly lipid accumulation induced by oxidized low-density lipoprotein [46].

### 2.4. Functional Enrichment Analyses of miRNA Target Genes

**Figure 3:**
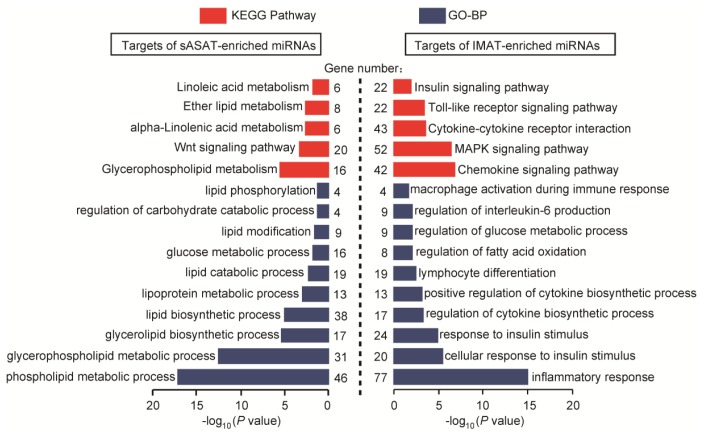
KEGG pathways and gene ontology biological process (GO-BP) categories enriched in the target genes of the top eight sASAT- and IMAT-enriched miRNAs. GO-BP is the GO terms under the biological process ontology.

To further highlight the distinct functional features of IMAT and sASAT, the target genes of the top eight DE miRNAs enriched in sASAT (967 mRNA genes) and IMAT (1707 mRNA genes) were predicted using PicTar [47], TargetScan human 6.2 [48] and MicroCosm Targets (version 5.0) [49] (Supplementary Table S6), and analyzed using DAVID [50] to determine whether or not they were enriched for specific functional categories and pathways. Similar to the finding for the DE miRNAs, the target genes of the IMAT-enriched miRNAs were primarily associated with inflammatory and diabetes-related processes, such as “inflammatory response” (77 genes, *p* = 1.21 × 10^−15^), “cellular response to insulin stimulus” (20 genes, *p* = 4.14 × 10^−6^), “lymphocyte differentiation” (19 genes, *p* = 3.75 × 10^−3^), “regulation of interleukin-6 production” (9 genes, *p* = 1.17 × 10^−2^), “macrophage activation during immune response” (4 genes, *p* = 2.74 × 10^−2^), “chemokine and toll-like signaling pathways” (42 genes, *p* = 1.61 × 10^−7^) and “insulin signaling pathway” (22 genes, *p* = 1.51 × 10^−2^). In contrast, the target genes of the sASAT-enriched miRNAs were mainly associated with lipid and energy metabolism, such as “glycerophospholipid metabolic process” (46 genes, *p* = 5.10 × 10^−18^), “lipid biosynthetic process” (38 genes, *p* = 7.36 × 10^−6^), “glucose metabolic process” (16 genes, *p* = 1.53 × 10^−2^) and “Wnt signaling pathway” (20 genes, *p* = 5.27 × 10^−4^) (Figure 3). These results further suggested that while sASAT is mainly involved in metabolic homeostasis, IMAT is susceptive to inflammation and should be regarded as a potential metabolic risk factor.

## 3. Experimental Section

### 3.1. Animals and Sample Collection

Three 210-day-old female Landrace pigs with normal weight (111.67 ± 1.15 kg) were used. The piglets were weaned simultaneously at 28 ± 1 day of age. A starter diet provided 3.40 Mcal·kg^−1^ metabolisable energy (ME), 20.00% crude protein and 1.15% lysine from the thirtieth to sixtieth day after weaning. From the 61st to the 120th day, the diet contained 3.40 Mcal·kg^−1^ ME, 17.90% crude protein and 0.83% lysine. From the 121st to 210th day, the diet contained 3.40 Mcal kg^−1^ ME, 15.00% crude protein and 1.15% lysine. The animals were allowed access to feed and water *ad libitum* and lived under the same normal conditions.

The macroscopic IMAT were directly separated from the regions that were beneath the biceps femoris muscle fascia of porcine hind leg. Since IMAT preparation could be easily contaminated by its surrounding tissues, we paid maximum attention to eliminate the others especially, such as connective tissue and muscle tissue, and all samples were resected from central part of tissue block. The sASAT were from the subcutaneous tissue of central abdomen near the last rib. All samples were immediately frozen in liquid nitrogen and stored at −80 °C before total RNA extraction.

### 3.2. Small RNA Libraries Construction and High-throughput Sequencing

Total RNA was extracted using the *mir*Vana™ miRNA isolation kit (Ambion, Austin, USA) following the manufacturer’s protocol. The integrity of total RNA was also tested via analysis by Bioanalyzer 2100 and RNA 6000 Nano LabChip Kit (Agilent, Palo Alto, CA, USA) with RIN number >6.0.

For a certain adipose tissue, equal amounts (5 μg) of total RNA isolated from three pigs were mixed. Approximately 15 μg of small RNA-enriched total RNA was prepared for Illumina sequencing. In general, the processing by Illumina consisted of the following successive steps: the small RNA ranged from 14 to 40 nt were purified by polyacrylamide gel electrophoresis (PAGE) and ligated specific adapters followed by polyacrylamide gel purification. Then the modified small RNA was reverse transcripted and amplified by RT-PCR. Finally, the enriched cDNA was sequenced on Genome Analyzer Instrument (GAI, Illumina, San Diego, CA, USA). The small RNA-sequencing data discussed in this publication have been deposited in NCBI’s Gene Expression Omnibus and are accessible through GEO Series accession number GSE30334.

### 3.3. Analysis of Small RNA-Sequencing Data

The raw reads were processed using Illumina’s Genome Analyzer Pipeline software and subsequently handled as described by Li *et al*. with some improvement [23]. After trimming off the sequencing adapters, the resulting reads was successively filtered by read length (only the read with the of 14 to 27 nt were retained), sequence component (containing <80% A, C, G or T; containing no more than two N (undetermined bases)) and copy numbers (the low-abundance reads (only the read with >3 counts were retained). Then the retained reads were searched against the NCBI [51], Rfam [52] and Repbase database [53] to remove porcine known classes of RNAs (*i.e.*, mRNA, rRNA, tRNA, snRNA, snoRNA and repeats). The sequencing reads survived from above strict filter rules were deemed as “high-quality reads”.

The high-quality reads were mapped to the pig genome (Sscrofa9) using NCBI Local BLAST following five steps in order: (1) map the high-quality reads to the 228 known porcine pre-miRNAs (encoding 257 miRNAs) and then to 6716 known pre-miRNAs (encoding 7952 miRNAs) from 24 other mammals in miRBase 18.0; (2) map the mapped high-quality reads to pig genome to obtain their genomic locations and annotations in Ensembl release 59 (Sscrofa 9, April 2009); (3) cluster the unmapped sequences in step 1 that mapped to the pig genome as putative novel miRNAs; and (4) predict hairpin RNA structures of the high-quality reads in step 3 from the adjacent 60 nt sequences in either direction from the pig genome using UNAFold [54]. To avoid ambiguous reads that have been assigned to multiple positions in pig genome, only reads longer than 18 nt in length were included in step 4.

### 3.4. miRNA Differential Expression Analysis

Program IDEG6 [33] was employed for detecting the DE miRNAs between two libraries. A unique miRNA is considered to be differentially expressed when it simultaneously obtains *p* < 0.001 under three statistical tests (a Audic-Claverie test, a Fisher exact test and a Chi-squared 2 × 2 test) with the Bonferroni correction.

### 3.5. Prediction and Functional Annotation of miRNA Target Genes

The potential targets of a certain miRNA were predicted by PicTar [47], TargetScan human 6.2 [48] and, MicroCosm Targets Version 5.0 [49], and the pairwise overlaps of results from three programs composed the final predicted targets. The predictions were according to the interactions of human mRNA-miRNA due to the absence of porcine miRNAs in current version of above-mentioned algorithm. The gene ontology biological process (GO-BP) terms and KEGG pathway terms enriched in predicted target genes were determined using a DAVID bioinformatics resources [50].

### 3.6. Q-PCR Validation

The expression changes of 14 selected miRNAs were validated by an EvaGreen-based High-Specificity miRNA qRT-PCR Detection Kit (Stratagene, La Jolla, CA, USA) on the CFX96™ Real-Time PCR Detection System (Bio-Rad, Hercules, CA, USA). The q-PCR validation were carried out on three biological replicates. The primer pairs were available in Supplementary Table S7. Three endogenous control genes (U6 snRNA, 18S rRNA and Met-tRNA) [23] were used in this assay. The ΔΔCt method was used to determine the expression level differences between surveyed samples. Normalized factors (NF) of three endogenous control genes and relative quantities of objective miRNAs were analyzed using the qBase software [55].

## 4. Conclusions

We have generated reliable miRNA transcriptomes of porcine sASAT and IMAT, and identified many known and novel miRNAs using small RNA-sequencing approach. We found that inflammationand diabetes-related miRNAs were enriched in IMAT compared with sASAT, which indicated the metabolic risk of the IMAT. A functional enrichment analysis of genes targeted by the enriched miRNAs also indicated that IMAT was mainly associated with the immune and inflammation response and may be a potential metabolic risk factor of obesity. The current study provides data that can be used in future studies to investigate the metabolic role of IMAT in obesity-related metabolic dysfunction. Our findings will also help promote the further development of the pig model for human metabolic research. It is also worth noting that further detailed comparision of IMAT between obese and non-obese individuals will be necessary and beneficial to decipher the role of miRNAs in adipogenesis and IMAT-related metabolic diseases.
